# Degenerated Cones in Cultured Human Retinas Can Successfully Be Optogenetically Reactivated

**DOI:** 10.3390/ijms21020522

**Published:** 2020-01-14

**Authors:** Sizar Kamar, Marcus H. C. Howlett, Jan Klooster, Wim de Graaff, Tamás Csikós, Martijn J. W. E. Rabelink, Rob C. Hoeben, Maarten Kamermans

**Affiliations:** 1 Netherlands Institute for Neuroscience, 1105 BA Amsterdam-Zuidoost, The Netherlands; sizarkamar@gmail.com (S.K.); mchowlett@gmail.com (M.H.C.H.); klooster.enkhuizen@gmail.com (J.K.); w.de.graaff@nin.knaw.nl (W.d.G.); t.csikos@amsterdamumc.nl (T.C.); 2 Department of Ophthalmology, Leiden University Medical Center, P.O. Box 9600, 2300 RC Leiden, The Netherlands; 3 Department of Cell and Chemical Biology, Leiden University Medical Center, P.O. Box 9600, 2300 RC Leiden, The Netherlands; m.j.w.e.rabelink@lumc.nl (M.J.W.E.R.); r.c.hoeben@lumc.nl (R.C.H.); 4 Department of Biomedical Engineering & Physics, Amsterdam University Medical Center, Meibergdreef 9, 1105 AZ Amsterdam, The Netherlands

**Keywords:** cultured human retina, cone photoreceptors, optogenetics, retinitis pigmentosa, vision restoration

## Abstract

Biblical references aside, restoring vision to the blind has proven to be a major technical challenge. In recent years, considerable advances have been made towards this end, especially when retinal degeneration underlies the vision loss such as occurs with retinitis pigmentosa. Under these conditions, optogenetic therapies are a particularly promising line of inquiry where remaining retinal cells are made into “artificial photoreceptors”. However, this strategy is not without its challenges and a model system using human retinal explants would aid its continued development and refinement. Here, we cultured post-mortem human retinas and show that explants remain viable for around 7 days. Within this period, the cones lose their outer segments and thus their light sensitivity but remain electrophysiologically intact, displaying all the major ionic conductances one would expect for a vertebrate cone. We optogenetically restored light responses to these quiescent cones using a lentivirus vector constructed to express enhanced halorhodopsin under the control of the human arrestin promotor. In these ‘reactivated’ retinas, we show a light-induced horizontal cell to cone feedback signal in cones, indicating that transduced cones were able to transmit their light response across the synapse to horizontal cells, which generated a large enough response to send a signal back to the cones. Furthermore, we show ganglion cell light responses, suggesting the cultured explant’s condition is still good enough to support transmission of the transduced cone signal over the intermediate retinal layers to the final retinal output level. Together, these results show that cultured human retinas are an appropriate model system to test optogenetic vision restoration approaches and that cones which have lost their outer segment, a condition occurring during the early stages of retinitis pigmentosa, are appropriate targets for optogenetic vision restoration therapies.

## 1. Introduction

Losing one’s vision though either injury or disease is obviously a traumatic life experience with major quality-of-life consequences. Restoring vision to those who have lost it, even to a limited degree, is a major technical challenge where several decades of research are beginning to bear fruit. The etiology of vision loss largely determines the type and location of the restorative intervention. For example, gene therapy has been successful where well-defined genetic mutations underlie the loss of vision such as in the cases of Leber’s congenital amaurosis [1,2] and choroideremia [3]. In cases of complete retinal degeneration, stimulating higher visual areas like the visual cortex with electrical prosthesis have shown some limited success [4] and several devices are undergoing clinical trials (clinicaltrials.gov registration numbers NCT03344848, NCT02747589, NCT02983370). Intuitively, however, the best outcomes are likely to occur by intervening as early as possible in the visual pathway in order to engage all remaining downstream visual-processing mechanisms. Ideally, the restorative procedure(s) would occur at the earliest steps of vision, which is possible when vision loss results primarily from degeneration of the outer retina such as occurs with retinitis pigmentosa (RP).

RP is a genetically heterogeneous disease [5] affecting approximately 1.5 million people worldwide [6]. It manifests initially as nyctalopia as rod-photoreceptors degenerate, which is followed later by a progressive decline in daylight vision as cone-photoreceptor function is also lost eventually resulting in blindness [7]. At later stages when rods and cones are completely lost, extensive retinal remodelling occurs [7,8,9], starting first in the outer retinal layers and progressing slowly to the inner retina. Nevertheless, in patients with moderate and severe RP, 78–88% of inner nuclear layer cells are seemingly preserved [10]. This implies that at the various disease stages, different parts of the retinal network are relatively undisturbed and may still function. An alluring prospect arising from this situation is that at each stage of the disease, the greatest degree of retinal processing may be rescued/recruited by targeting restorative procedures towards the largely unaffected retinal layers. Restorative procedures could target dysfunction cones, sometime still present years after their outer segments are damaged [8,11], at earlier stages shifting to bipolar cells (BCs), and eventually ganglion cells (GCs), for increasingly advanced cases [12,13]. However, to be effective, such approaches require developing techniques that can deliver cell-type appropriate signals to large numbers of specifically targeted classes of retinal neurons.

Currently, the most developed techniques for restoring vision to RP patients are epiretinal, subretinal and suprachoroidal prostheses. While the exact details of each device differ somewhat, they are all essentially arrays of electrodes that electrically stimulate retinal cells in order to restore vision in blind patients [13]. However, the degree of restoration produced by these devices is still in the ultra-low vision range [14]. Indeed, recently the creators of the Alpha-IMS subretinal prosthesis chose to discontinue as after 16 years of research they were unable to show any benefit to patient’s everyday life. The impressive associated technical advancements notwithstanding, these modest outcomes are perhaps not unsurprising considering (a) the low number of electrodes these devices use (e.g., 60 in the current Argus II design) relative to the number of cells present in the human retina (e.g., approximately 1 million GCs [15]) and (b) each electrode presumably affects hundreds of cells [16], indiscriminately sending the same signal down multiple classes of retinal cell types that would normally each act as distinct feature-specific channels. Even if the technical issues associated with increased electrode density can be overcome, such as increased impedance [17] and electrical crosstalk [18], the issue of cell specificity remains.

In contrast, optogenetic approaches have the potential to restore vision by converting large numbers of specific cell types into light sensors, which can then be stimulated with cell-type appropriate signals. Optogenetic techniques photosensitize neurons by genetically introducing light-sensitive actuators like ion channels or pumps into the cell membrane using viral vectors where the promotor sequence used determines the specificity of the expression. Photosensitizing cones [19] or ON-BCs [20,21,22,23,24,25] in blind mice has restored several retinal visual-processing circuits including GC ON- and OFF-light responses, centre-surround receptive fields and direction selectivity as well as visually evoked responses in the dorsal lateral geniculate nucleus (dLGN) and visual cortex, optomotor responses, innate behavioural reactions to light increments, temporal and contrast modulated flickers and drifting grating with low spatial frequencies, and learned visually guided behaviours such as choice-decision tasks. Compared with non-targeted expression, restricting expression to ON-BCs increases the restored response-type diversity of dLGN neurons and expands the range of visual stimuli that elicited a behavioural response, including one indicative of an intact and functional retinal looming circuit [23]. As non-targeted expression will cause many different cell types, which normally have diverse visual feature selectivity, to have the same light response some reduction in outcomes are to be expected. Similarly, bypassing earlier retinal processing and targeting the GCs would also limit the extent of possible restoration. Despite this, non-targeted and GC-targeted expression is still able to achieve a considerable degree of restoration. GC firing to light stimuli flows through to the visual cortex and an impressive amount of visual acuity and contrast sensitivity when measured by optomotor responses has been restored in mice as have light avoidance, freezing and object recognition behaviours [26,27,28,29,30,31,32,33,34], but see also [35]. Targeted expression has also photosensitized GCs in macaque and cultured human retina, enabling them to response directly to light stimuli [27,28]. 

Thus far, the potential of optogenetic therapies to restore vision looks promising and there are currently two phase I/IIa human clinical trials underway (clinicaltrials.gov registration numbers NCT02556736, NCT03326336). However, optogenetic restoration is still far from a panacea as there are several major drawbacks to overcome. For instance, the commonly used light-sensitive actuator, Channelrhodopsin-2, requires dangerously high levels of blue light to activate it [27,28] and most light-sensitive actuators lack a signal amplification cascade so in order to induce a meaningful effect their expression levels in a cell’s membrane must be high. Approaches addressing these issues, including red-shifting the channelrhodopsin to spectral sensitivities, were the high intensities required for activation that can be safely used [27], reducing the channelrhodopsin deactivation kinetics to increase its operation light sensitivity [29], using actuators with signal amplification mechanisms such as the G-protein coupled rod opsin [23], and engineering vectors with better tissue penetration [36] or more efficient promotor sequences [28] to increase transgene expression. However, the efficacies of optogenetic therapies utilizing improved actuator biophysical-properties and vector-delivery systems may differ between model systems and humans [37]. Thus, a human model system working in conjunction with animal models would aid the development of optogenetic therapies. 

We cultured post mortem human retinas for up to 10 days using the protocol described by Busskamp et al. [19] and investigated the effectiveness of activating cones optogenetically. Here, we ask three questions: (1) Are the cones in these cultured human retinas still viable neurons? (2) Can they be optogenetically reactivated? (3) Are their synaptic connections still functional? We found that cones lose their outer segments and thus their light sensitivity during the first days of culturing. The cultured cones still expressed ion channels typical of those found in other vertebrate species with one exception—they sometimes had a fast sodium current. When transfected with halorhodopsin, cultured cones displayed light responses that were transmitted to second order neurons such as horizontal cells (HCs), as evidenced by the feedback responses we recorded in the transduced cones. Finally, we could demonstrate that the restored light responses of cones were transmitted to GCs. These results show that the cultured human retina is an appropriate model system to study optogenetic vision restoration strategies and that cones are a suitable target.

## 2. Results

### 2.1. Immunohistochemistry

To ensure that our culture preparations were in good shape, we qualitatively assessed the retina’s overall structure using several immunocytochemical markers at four different time points: Immediately prior to (T0), and after 4 (T4), 7 (T7) and 10 (T10) days of, culturing. In each case, we examined 12 retinal sections obtained from two donor eyes.

**Figure 1:**
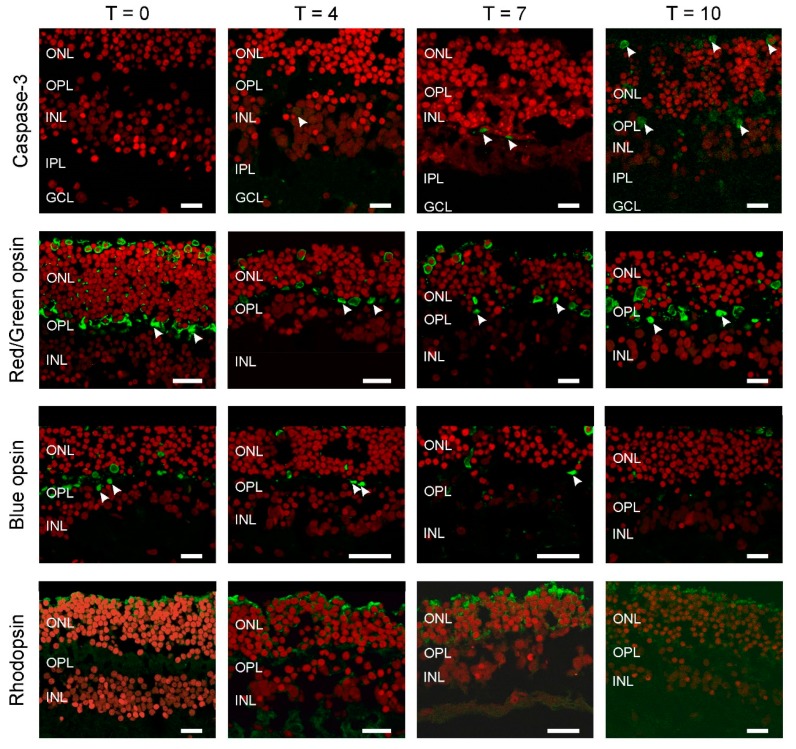
All photoreceptors have lost their outer segments. Apoptosis was studied by Caspase-3 labelling (**top row**). The number of apoptotic cells (arrowheads) remains very limited even after 10 days of culturing (T10). Cone (**middle rows**), rod (**bottom row**) and opsin expression occurred throughout the photoreceptors. Round somata of the photoreceptors are visible at the upper layer of the retina as round structures. An axon connects the somata with the synaptic terminals (arrowheads) in the outer plexiform layer (OPL). The red label is the nuclear stain ethidium bromide. (ONL: outer nuclear layer; INL: inner nuclear layer; IPL: inner plexiform layer; GCL; GC layer. Scale bars indicate 10 µM.

To estimate the extent of cell death occurring with increased culturing time we used a marker for apoptosis, cleaved Caspase-3 (Figure 1; Green: Caspase-3; Red: ethidium bromide). We did not see any labelling at T0 and although the numbers of labelled cells increased with longer culturing times the overall numbers remained low. We found one labelled cell at T4 (Figure 1, arrowhead), increasing to two and five at T7 and T10, respectively (Figure 1, arrowheads). This illustrates that large-scale cell death did not occur during the first ten days of culturing. The apoptotic cells found were in either the outer nuclear layer (ONL) and inner nuclear layer (INL).

Next, we determined if all the photoreceptor types remain present during culturing and if so where they are located (Figure 1). At all time-points, the expression of rhodopsin, red/green and blue opsin occurred and was restricted to large round somata at the top of the ONL with axons going down all the way to the outer plexiform layer (OPL), where they formed synaptic contacts with second order neurons (arrowheads). In addition, all labelled photoreceptors lacked an outer segment. 

**Figure 2:**
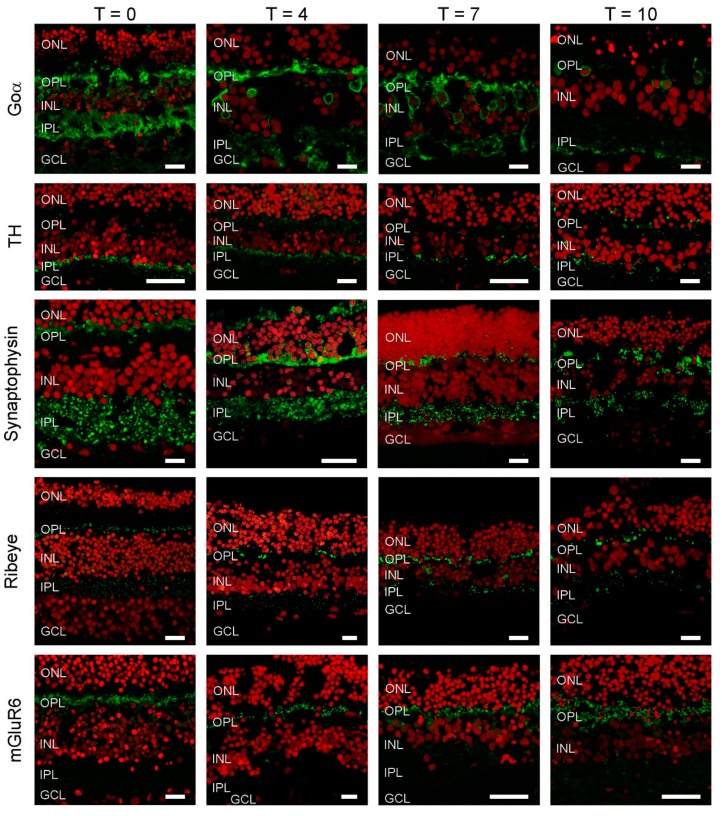
Cellular markers indicate that retinal morphology is relatively undisturbed. Goα (green), a marker for ON-bipolar cells, labels cells in the INL with dendritic processes extending into the OPL and big axon terminals in the IPL. At T10, labelling seems less apparent. Tyrosine hydroxylase (TH) (green), a marker of dopaminergic amacrine cells shows an intense band of labelling at the border of the INL and IPL with some puncta in the OPL. This labelling pattern does not change dramatically during culturing. Synaptophysin (green), a synaptic vesicle marker shows strong labelling in both plexiform layers. The labelling pattern remains relatively stable during the 10 days of culturing. Ribeye (green), a marker for ribbon synapses is present in both plexiform layers. Note the horseshoe shaped structures in the OPL, which are indicative of cone synaptic terminals. The labelling pattern remains relatively stable during the 10 days of culturing. mGluR6 is a marker for the photoreceptor to ON-bipolar cell synapse. Strong labelling in the OPL remains throughout the whole culturing period. Red: Nuclear stain ethidium bromide. Scale bar represents 10 µM.

Immunostaining for the ON-BC label, Go_α_ (Figure 2), was present in BC somas high in the INL, in BC dendrites and in the inner plexiform layer (IPL). This staining pattern indicates the retinal architecture of ON-BCs is retained during culturing, at least for the first 7 days. After this point, labelling becomes far sparser. Tyrosine hydroxylases (TH) immunolabelling was largely restricted to cell bodies and processes in the proximal IPL during the first 7 days of culturing. This suggests the structural organization of dopaminergic amacrine cells remains preserved during culturing. However, by T10, labelling appears more distributed within the IPL, indicating some remodeling has occurred.

We also investigated if the synaptic structures between cells were still intact. At all time-points, synaptophysin labelling indicated that synaptic vesicles were present in both the IPL and OPL. Similarly, ribeye labelling showed that BC and photoreceptor synaptic ribbons were also present in these layers, respectively. Labelling of the metabotropic glutamate receptor (mGlur6), typically present only on the dendrites of ON-BCs, was restricted to the OPL. Combined these results shows that the architecture of the retina, and the machinery and organization of the retinal synapses, remains in relatively good shape even after 7 days of culturing. 

### 2.2. Electrophysiology

**Figure 3:**
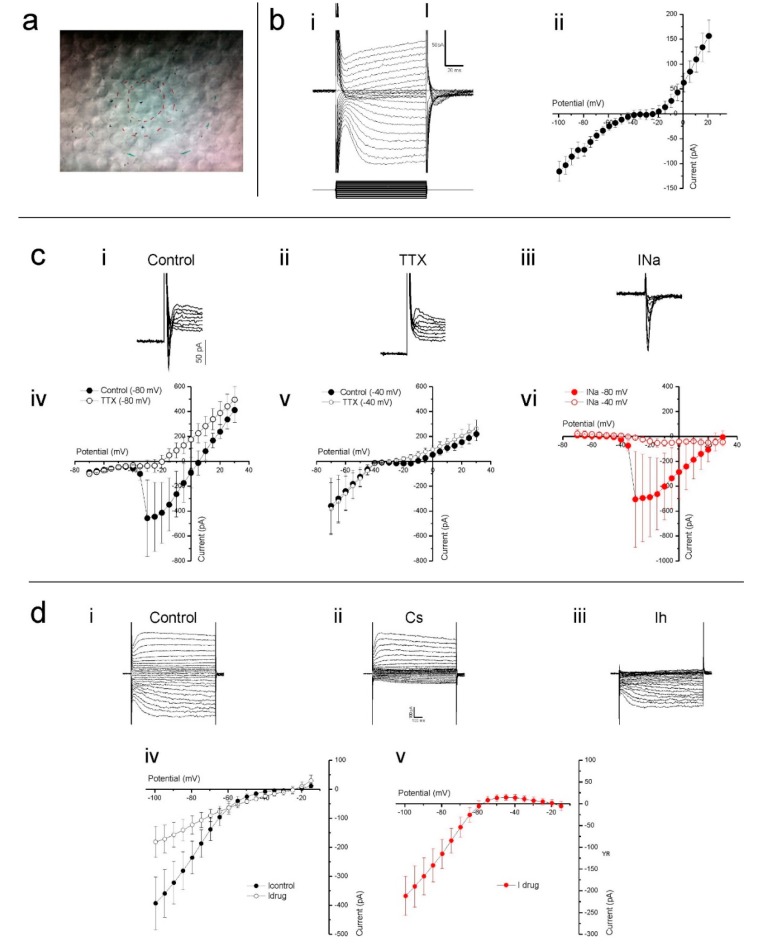
Cones in cultured human retinas display several prominent ionic conductances. (**a**) The cone cell bodies were typically round in shape and located on the explant surface, as most photoreceptor outer segments were either missing or severely degenerated. The red inner circle indicates the stimulus spot of 20 µm. The other marks are other alignment references. (**b**) Slowly activating inward and outward currents are readily distinguishable when voltage clamping cones at −40 mV and stepping them to potentials ranging from −100 mV to +30 mV, as demonstrated by the example given in (**b**i). When plotted as an IV-relation (**b**ii), a non-linearity between −45 mV to −15 mV is also apparent. (**c**) Clamping cones at −80 mV then stepping them to potentials above −40 mV often elicited a fast and transient inward current (**c**i). This current did not occur in the presence TTX (**c**ii, iv), a potent sodium channel blocker, nor when cones were held at −40 mV then stepped to more depolarizing potentials (**c**v). The control-TTX subtracted sodium current (**c**iii) IV-relation (**c**vi) further demonstrates that the current only activates if cones are initially very hyperpolarized before moving to more depolarized potentials. (**d**) Stepping cones to hyperpolarized potentials from −40 mV induced a slowly activating inward current that CsCl blocked, as both the whole cell currents (**d**i,ii) and IV-relations (**d**iv) demonstrate. The control-CsCl subtracted whole cell current (**d**iii) and corresponding IV-relation (**d**v) bare all the characteristics associated with a hyperpolarization-activated current. Data shown as mean ± SEM in bii (*n* = 12), civ-vi (*n* = 5) and div, v (*n* = 6).

Having established that the cultured human retina is structurally in good condition, we assessed the viability of the cones that lost their outer segment at an electrophysiological level by studying the properties of several known cone ionic currents. To do so, whole-cell voltage-clamp recordings were made from cones in flat mounted cultured human retinas. In such preparations, cones are round in shape and located on the retinal surface (Figure 3a). Cones were voltage clamped at a holding potential of −40 mV and stepped for 100 ms to potentials ranging from −100 mV to +20 mV (Figure 3b). In the example cone’s current response, shown in Figure 3(bi), slowly activating inward and outward currents are readily distinguishable. Figure 3(bii) shows the mean (±SEM) IV-relation of 12 cones. Here, at positive and negative potentials, the IV-relation is rather linear, whereas a strong non-linearity occurs between around −45 to −15 mV, which is most likely generated by a voltage gated Ca^2+^ current (I_Ca_). 

Occasionally, we found a fast-transient inward current. To test if this was a fast-transient Na^+^ current (I_Na_), we clamped nine cones at both −80 and −40 mV and stepped them to a maximum of +30 mV in 5 mV increments. When stepped from a holding potential of −80 mV (Figure 3(ci)), five of the nine cells showed a fast inward current, whereas when stepping from a potential of −40 mV, this current only occurred in one of the nine cells. This is consistent with the inactivation properties of I_Na_ [38]. Additionally, 1 µM tetrodotoxin (TTX) prevented the fast-transient inward current from occurring when stepped from a holding potential of −80 mV (Figure 3(cii,civ)) but it had no effect on cone currents when stepped from a potential of −40 mV (Figure 3(cv)). The control-TTX subtracted current shown in Figure 3(ciii,cvi) indicates that when held at −80 mV, the fast transient TTX sensitive inward current begins to activate when stepped to −35 mV and peaked when stepped to −30 mV. 

From the membrane currents shown in Figure 3(bi), it is immediately apparent that hyperpolarizing voltage steps induce a slowly activating inward current. This current profile is characteristic of the hyperpolarization-activated inward current (I_h_), which is commonly observed in vertebrate cones [39,40,41]. To confirm that this was the *h* current, and determine its IV-relation, cones voltage clamped at −40 mV were stepped from −100 to +20 mV in 5 mV increments prior to and during the presence of 1 mM CsCl, a I_h_ blocker. CsCl greatly reduced the slow inward currents evoked by the hyperpolarizing command steps, an effect equally apparent when comparing either individual whole cell currents (Figure 3(di,dii)) or the mean (±SEM, *n* = 6) IV-relations (Figure 3(div)) measured in both conditions. The CsCl sensitive current, obtained by subtracting the drug-condition whole-cell currents from those occurring during the control condition, activated slowly upon hyperpolarization (Figure 3(diii)) and steadily increased in size with increased hyperpolarization from approximately −55 mV as demonstrated by its IV-relation (Figure 3(dv)). These characteristics identify this current as I_h_.

**Figure 4:**
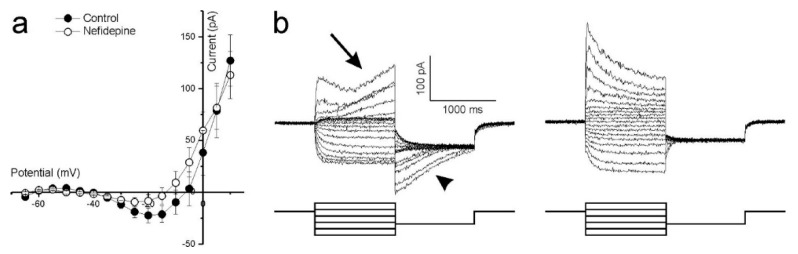
Ca^2+^-dependent conductances in cultured human retina cones. (**a**) The mean (±SEM, *n* = 5) leak-subtracted IV-relation of cones voltage clamped at −60 mV and stepped to potentials ranging from −100 to +20 mV. The inward current activating around −40 mV and peaking around −20 mV is reduced (*p* = 0.022) in amplitude by nifedipine, an L-type Ca^2+^ channel blocker. (**b**) Whole cell currents of a representative cone, clamped at −40 mV then stepped to potentials ranging from −80 mV to +20 mV for 1 s before being stepped back to −60 mV for 1 s. The slowly developing outward current occurring at more depolarized potentials (left-arrow) and the associated dissipating current when these potentials are stepped back to −60 mV (left-arrowhead) are both absent in the presence of the Ca^2+^-dependent Cl^−^-current blocker, niflumic acid (right).

Ca^2+^ influx controls photoreceptor glutamate release, hence an intact I_Ca_ is crucial if optogenetically reactivated cones are to transmit information to second order retinal neurons. The non-linearity present between −45 and −15 mV in the whole-cell current IV curve shown in Figure 3(bii) suggests strongly that cones in cultured human retinas have a functional I_Ca_. To confirm this, cones were stepped from −100 to +20 mV in 5 mV increments from a holding potential of −60 mV. The mean (±SEM, *n* = 5) IV-relation (Figure 4a, closed circles) shows an inward current activating around −40 mV and increasing steadily in size until reaching a peak around −20 mV. At more depolarized potentials, the IV curve flips to a positive slope and the current decreases in magnitude as the command potential become more depolarized. These characteristics are consistent with the I_Ca_ found in vertebrate photoreceptors [42,43,44,45], which is mediated by voltage sensitive L-type Ca^2+^ channels. Adding 10 μM of the dihydropyridine L-type Ca^2+^ channel blocker, nifedipine, reduced the current’s peak amplitude on average by 55 ± 6.6% (*p* = 0.022, Figure 4a, open circles) confirming that it is a L-type Ca^2+^ channel mediated I_Ca_.

Cones in other vertebrate retinas normally express a large Ca^2+^-dependent Cl^−^-current (I_Cl(Ca)_) [46], whose activation depends on the intracellular Ca^2+^-concentration. As cone intracellular Ca^2+^ concentration dynamics are relatively slow [46], a characteristic feature of the cone I_Cl(Ca)_ is its slow activation and inactivation time constants. Cones in the cultured human retinas also display currents with these features as Figure 4b (left) shows. Here, cones were stepped from a −40 mV holding potential to values between −80 mV and +20 mV for 1000 ms to give I_Cl(Ca)_ time to develop. The potential was then stepped to −60 mV to inactivate I_Ca_ and held for 1000 ms to give I_Cl(Ca)_ time to discharge. At more depolarized potentials where I_Ca_ is near maximal, an outward current starts slowly developing approximately 300 ms after the onset of the command potential (arrow). When these potentials step back to −60 mV, a slowly dissipating tail-current occurs (arrowhead). Neither of these two slow currents occurred in the presence of 100 μM niflumic acid, an I_Cl(Ca)_ blocker (Figure 4b, right). 

The combined electrophysiological results suggest that cultured human retina cones are functionally intact with the expected ionic conductances.

### 2.3. Optogenetic Restoration of Light Sensitivity in Cones

Both the immunohistochemistry and electrophysiological outcomes indicate that when cultured the post-mortem human retina is in good condition. Since all cones lost their outer segments during culturing, we never observed a light response. Next, we aimed at restoring light sensitivity to these cones using optogenetics, by transducing into their membranes the light-gated Cl^−^ pump, eNpHR. Light stimulation should induce an influx of Cl^−^ ions causing the cones to hyperpolarize, which mimics the ‘normal’ vertebrate cone light response.

**Figure 5:**
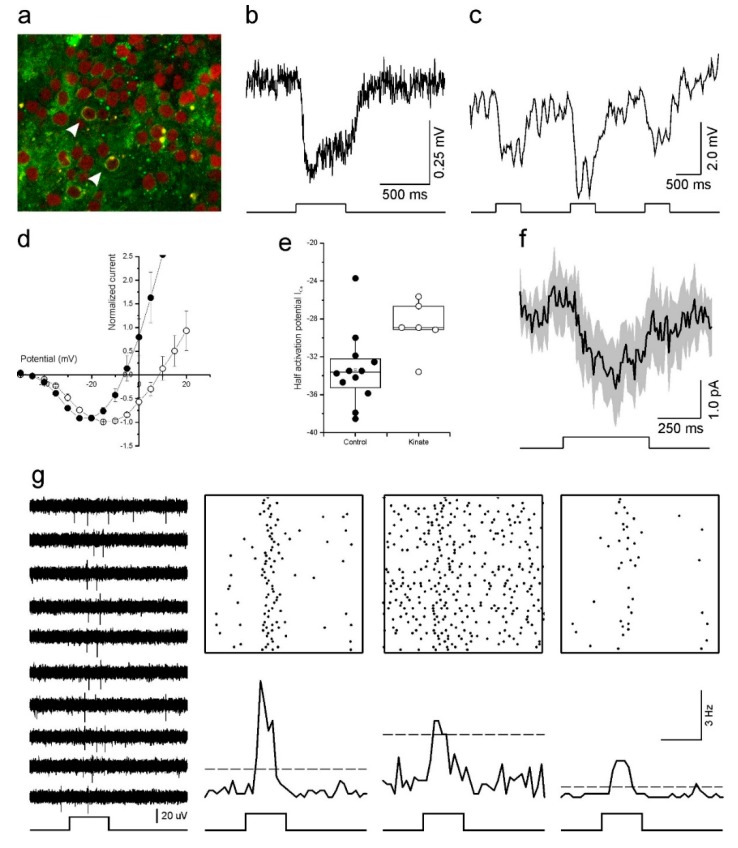
Optogenetic reactivation of cultured human retinas. (**a**) Fluorescent image of a transfected retina. Arrowheads indicate transfected cones with eNpHR-EYFP in their membrane (green). The red label is a nuclear stain (ethidium bromide). (**b**) The light response of an optogenetically reactivated cone to a full-field light-flash stimulus. The restored response begins soon after the onset of the 500 ms light stimulus (bottom) and remains for the duration of the flash. (**c**) An optogenetically reactivated cone’s light response to 500 ms flashes of blue (465 nm), green (525 nm) and red (624 nm) LED light. The flash of green light elicited the largest hyperpolarizing response. (**d**,**e**) Depolarizing horizontal cells with kainic acid shifted the cone I_Ca_ voltage dependency to more positive potentials as demonstrated by the mean (±SEM) leak subtracted and normalized IV-relations (**d**) and I_Ca_ half activation potential (**e**) for cones in control conditions (closed circles, *n* = 12) and for cones treated with 30 μM kainic acid (open circles, *n* = 6). (**f**) The light-induced horizontal cell feedback signal measured in cones (mean ± SEM, *n* = 7). The feedback signal is evident by the inward current deflection occurring in conjunction with the full-field light flash. (**g**) Multi-electrode array recordings of light induced ganglion cell responses from a retina with optogenetically reactivated cones. Representative traces of extracellular potentials from one electrode to a repeated 500 ms full-field light flash stimulus are shown on the left. The upper right panels show Rasta plots of spiking activity occurring on three electrodes during one hundred repetitions of the full-field light flash stimulus. The same data is redrawn as peristimulus time histograms (50 ms bin widths) in the lower right panels, where the dashed lines indicate the mean +5 standard deviations firing rate that occurred prior to the flash onset. In both representations increased ganglion cell firing coincides with the light stimulus. Optogenetic reactivation of cultured human retinas. (**a**) Fluorescent image of a transfected retina. Arrowheads indicate transfected cones with eNpHR-EYFP in their membrane (green). The red label is a nuclear stain (ethidium bromide). (**b**) The light response of an optogenetically reactivated cone to a full-field light-flash stimulus. The restored response begins soon after the onset of the 500 ms light stimulus (bottom) and remains for the duration of the flash. (**c**) An optogenetically reactivated cone’s light response to 500 ms flashes of blue (465 nm), green (525 nm) and red (624 nm) LED light. The flash of green light elicited the largest hyperpolarizing response. (**d**,**e**) Depolarizing horizontal cells with kainic acid shifted the cone I_Ca_ voltage dependency to more positive potentials as demonstrated by the mean (±SEM) leak subtracted and normalized IV-relations (**d**) and I_Ca_ half activation potential (**e**) for cones in control conditions (closed circles, *n* = 12) and for cones treated with 30 μM kainic acid (open circles, *n* = 6). (**f**) The light-induced horizontal cell feedback signal measured in cones (mean ± SEM, *n* = 7). The feedback signal is evident by the inward current deflection occurring in conjunction with the full-field light flash. (**g**) Multi-electrode array recordings of light induced ganglion cell responses from a retina with optogenetically reactivated cones. Representative traces of extracellular potentials from one electrode to a repeated 500 ms full-field light flash stimulus are shown on the left. The upper right panels show Rasta plots of spiking activity occurring on three electrodes during one hundred repetitions of the full-field light flash stimulus. The same data is redrawn as peristimulus time histograms (50 ms bin widths) in the lower right panels, where the dashed lines indicate the mean +5 standard deviations firing rate that occurred prior to the flash onset. In both representations increased ganglion cell firing coincides with the light stimulus.

Transducing the cones with LV-HXARR3-eNpHR-EYFP resulted in the membranes of many cone somas expressing eNpHR-EYFP (Figure 5a). These cones also responded when stimulated with light (Figure 5b). Here, EYFP positive cones were current clamped and stimulated with a full-field flash of white light for 500 ms. The ensuing hyperpolarizing membrane potential was sustained for the duration of the light flash, rapidly returning to baseline at light offset. The chromatic sensitivity of the light response matched that of eNpHR, as light flashes from a 525 nm LED induced the largest hyperpolarizing response whereas responses were smaller to flashes from a 465 nm or a 624 nm LED (Figure 5c). 

### 2.4. Restoration of Synaptic Transmission

We next asked if the restored light response of cones flows downstream to other retinal neurons. The immunohistochemistry suggests the outer retinal synapse is morphologically intact and the electrophysiology shows the cone current needed for vesicle release, I_Ca_, is also functional. However, can cones in cultured human retina still transmit their restored light response to subsequent retinal neurons?

To begin addressing this issue we first examined the HC to cone feedback signal. HCs provide negative feedback to cones by modulating the cone I_Ca_ [42]. When HCs hyperpolarize, either by light stimulation or pharmacologically, the resultant feedback signal is manifest in the cone I_Ca_ as a shift in its voltage activation towards more negative potentials [42]. Conversely, when HCs depolarize their feedback signal shifts the cone I_Ca_ voltage dependency towards more positive potentials [47]. To test if HC feedback was present in our cultured human retinas, we compared the cone I_Ca_ voltage dependency in control conditions and when 30 µM kainic acid (KA), a glutamate receptor agonist, was present. If HCs are still functional and sending a feedback signal to cones, then depolarizing HCs with KA should shift the cone I_Ca_ voltage dependency towards positive potentials. As Figure 5d shows, this was indeed what we found. When KA was present (*n* = 6), the cone I_Ca_ peak occurred at potentials 6.7 ± 1.23 mV (*p* = 0.025) more positive than for cones in the control condition (*n* = 12). Similarly, the I_Ca_ half activation potential (Figure 5e) also occurred at more positive potentials in the KA condition (−28.8 ± 1.12 mV) compared to controls (−33.3 ± 1.11 mV, *p* = 0.022). 

Having found that the HCs are functional, and the HC to cone synaptic pathway still operating, we next asked if we could measure a light induced feedback signal in cones after transducing the retina with halorhodopsin. To do this, cones were voltage clamped at −40 mV and the light response of the recorded cone was saturated by a bright 20 µm spot of white light. Then a full-field white light stimulus was flashed on for 500 ms to stimulate all other cones with a restored light response, and consequently hyperpolarize HC. In this paradigm, a HC feedback signal is evident by an inward current deflection occurring in conjunction with the full-field light flash. Figure 5f shows the averaged (±SEM) response of seven cones under this stimulus and a small inward current deflection does occur during the full-field stimulation, indicating that the cones received a light mediated HC feedback signal. This result suggests that in our human retina cultures (a) resensitized cones responded to the light flash stimulus and were able to transmit this information to postsynaptic neurons, (b) enough cones were resensitized to sufficiently hyperpolarize HCs to the extent that they generated a feedback signal, and (c) this HC feedback signal was fed back to the cones and modulated their I_Ca_.

If synaptic communication is restored at the outer retinal synapse, will the signal continue to flow down to the GCs? To test this, we switched to multielectrode arrays (MEAs). In the three transduced retinal pieces we recorded from using MEAs, we generally saw very little spontaneous GC activity. However, in one of the three pieces, we recorded GC spiking activity indicative of ON-light responses (Figure 5g). The light responses were relatively slow-onset and transient. The firing rate initially increased, quickly reached a peak before declining back towards background levels.

## 3. Discussion

This study validates the cultured human retina as a viable model system with which to study optogenetic vision restoration approaches. We showed immunohistochemically that the retinas remain in relatively good condition during the first 7 days of culturing. With electrophysiological approaches, we further showed that cones in cultured human retinas express the expected ionic conductances and that transfecting them with halorhodopsin can restore their light responses. These reactivated cones were able to drive HCs such that they could in turn send a light mediated feedback signal back to the cones. Finally, we showed evidence that the restored cone light responses could drive GCs responses. Given these results, we believe that cultured human retinas are a highly useful preparation for testing and refining optogenetic tools aimed at restoring vision. Moreover, achieving a broad restoration of cone light responses may even allow fundamental biophysical human retinal processing to be studied.

### 3.1. Morphological Condition of Cultured Human Retinas

In general, the overall structure of the cultured human retina stays intact during culturing, suggesting that the architecture, and synaptic organization remains in relatively good shape even after more than 7 days of culturing. However, we did find that cones lost their outer segments almost immediately after the start of culturing. In addition, cones sometimes became apoptotic after some days of culturing. This is not surprising considering photoreceptors were missing their outer segments as well as their contact with the retinal pigment epithelium, which plays an important role in photoreceptor survival [48]. Even so, the number of apoptotic cells remained surprisingly low and even at T10 there was no indication that large-scale cell death occurred, indicating that our culturing conditions were reasonably good. 

Our results are similar to those of Fernandez-Bueno et al. [49], who found that cultured human retinas start showing morphological changes after 9 days of culturing. However, they found synaptophysin had become mislocated by day 9 of culturing whereas we saw no such change. Others have shown that organotypic retinal cultures slowly and progressive degenerate, with deterioration becoming increasingly more evident after culturing periods of 3 days (human: [49,50,51] and mouse: [52,53]). The reason for these discrepancies presumably lays in the differing culturing conditions and tissue preparation procedures used. Continued optimization of the culturing conditions promises to extend the period retinas can be maintain, with Szabo, et al. [54] managing to keep retinas in good condition for about 21 days.

### 3.2. Ion-Channel Makeup of Cones in Cultured Human Retinas

Occasionally, we found I_Na_ in cones (Figure 3c). This current was not expected as there are no reports of I_Na_ occurring in cones from acutely isolated retinas from any other vertebrate studied [55] and photoreceptors do not generate action potentials. However, I_Na_ has previously been shown in freshly dissociated human cones [56] and in the rods of human retinal explants from retinal detachment patients [57]. The dissociated cones also lacked an outer segment. Possibly cones start to express I_Na_ when experiencing stress. The presence of I_Na_ in degenerated cones may interfere with their optogenetic reactivation, since the current could make the cone membrane potential less stable. On the other hand, the presence of I_Na_ may not be an important confounder considering its activation requires hyperpolarized membrane potentials well outside the normal working range of cones. Regardless, possible effects arising from the aberrant expression of I_Na_ in degenerated cones requires attention. 

Except for the presence of I_Na_, the ion-channel makeup of cones in cultured human retina was strikingly similar to those found for non-human vertebrate cones in freshly isolated retina. This indicates that the culturing procedure does not affect the major electrophysiological properties of the cones. The Ca^2+^ current (Figure 4a) could be partially blocked with 10 µM nifedipine and peaked around −20 mV, confirming that it was an L-type Ca^2+^ channel. This was expected as cones in all other vertebrates studied express such Ca^2+^ channels [58,59,60,61,62]. The presence of a functional cone I_Ca_ in the cultured human retina is encouraging as Ca^2+^ influx though these channels modulate the release of glutamate. As far as we know, this is the first electrophysiological report of I_Ca_ in human photoreceptors. 

Apart from regulating the release of glutamate, Ca^2+^ influx into cones also modulates I_Cl(Ca)_ [46]. Indeed, the I_Cl(Ca)_ is typically one of the largest photoreceptor currents and is characterized by its slow activation and inactivation times but its function still remains elusive. We also found that cultured human retina cones have such a current (Figure 4b). Concomitant with the slow development of I_Cl(Ca)_ during a prolonged stepping protocol, large slowly dissipating tail currents occurred on return to −60 mV. Both the slowly developing I_Cl(Ca)_ and the tail currents were blocked by 100 µM niflumic acid. As far as we are aware, this is the first demonstration of I_Cl(Ca)_ in human cones.

We also observed I_h_ (Figure 3d), which is another major current commonly observed in vertebrate cones such as from goldfish [63], salamander [40], monkey [64] and human [65]. This current plays an important role in shaping the photoreceptor light response by increasing the transientness of the response, an effect similar to that of a high-pass filter [41]. It is also implicated in the adaptation of cones to contrast when processing natural scenes [63].

### 3.3. Reactivation of Cones

We were able to make the cultured cones light sensitive again by transducing them with LV-HXARR3-eNpHR-EYFP. However, their light responses (Figure 5b,c) were considerably smaller compared to those reported by Busskamp, et al. [66] in a similar preparation. The differences may result from lower expression levels in our experiments. Despite the small cone response amplitude, we were able to record HC induced feedback responses in cones (Figure 5f) and GC spiking activity (Figure 5g). However, the GC activity was rather weak. This left us questioning if these GC light responses were artefactual in origin, arising perhaps from crosstalk from the light stimulator or from an inherent light sensitivity of the MEA itself. We are confident however that these responses were real for the following reasons. Firstly, light responses were only seen on a limited number of electrodes. A number of other electrodes showed spontaneous activity but had no light responses. In contrast, inherent MEA photosensitivity, or cross talk from the light stimulator should appear across all recording electrodes. Secondly, any stimulus-related confound should coincide with the light-onset and remain for the duration of the light flash whereas we observed short-lived, slowly-activating transient responses. Thirdly, the light responses were relatively short lived, disappearing after approximately 10 min exposure to the light flash stimulus. Any non-biological signal should remain present. In summary, we are confident that the GC light responses are of retinal origin. However, we cannot discount the possibility that the light responses were from intrinsically photosensitive GCs, although the transient nature of their response would suggest otherwise [67,68]. 

HCs in the human retina seem to feed back to cones using a mechanism similar to that found in all other vertebrates studied so far [42]. Since we did not study the feedback pathway in detail we cannot comment on the exact mechanism underlying it, be it an ephaptic [69] or pH-buffering mediated system [70] or a combination of the two. However, given how similar the modulation of the cone I_Ca_ in human retina is with other well-studied vertebrate retinas, it is most likely that the underlying mechanism will be a combination of the two. It will be very interesting to study this further and determine the relative contributions of the two proposed mechanisms. 

### 3.4. Optogenetic Vision Restoration in RP

A crucial step in the development of optogenetic restoration of human vision is to test it in human retinas and determine whether the restored retinal processing is stable and how it compares to that of healthy retina. Ideally, we would like to do these tests in retinal explants from RP patients. However, such donor retinas are very rare. In early stages of RP, photoreceptors lose their outer segments but their somata remain present and are likely to still be synaptically connected to the rest of the retina. These characteristics are analogous to those we find when culturing retinas from human donors without known eye diseases; cones lose their outer segment but their somata remain present and synaptically connected to the rest of the retina. We further show that these photoreceptors are viable and can drive retinal processing in a rather normal way, but with the caveat that they sometimes acquire I_Na_. If, and how, disruptive the presence of this aberrant current is remains to be determined. Nevertheless, our present study indicates that the cultured human retina is a useful model to study optogenetic vision restoration approaches for retinal diseases like RP and that degenerated photoreceptors are an appropriate target for these strategies.

## 4. Materials and Methods

### 4.1. Human Eyes

Post-mortem human donor eyes from individuals without known eye diseases were obtained from the Euro Tissue Bank Amsterdam or the Netherlands Brain Bank. All parties received permission to perform autopsies, for the use of tissue for research purposes from the Euro Tissue Bank Amsterdam and the Netherlands Brain Bank. All donors had given informed consent for autopsy and use of their eye tissue for research purposes. The investigations were carried out following the rules of the Declaration of Helsinki of 1975 (https://www.wma.net/what-we-do/medical-ethics/declaration-of-helsinki/), revised in 2013. Details regarding each donor are given in Table 1.

### 4.2. Culturing

The anterior part of the eye was removed and the eye-cup transferred to a CO_2_-independent medium (Invitrogen, Carlsbad, CA, USA), 37 °C, supplemented with 1% Penicillin-Streptomycin-Glutamine and 2 mM L-glutamine. The retina was gently separated from the vitreous humor and the retinal pigment epithelium, cut into ~0.5 × 0.5 cm pieces, placed receptor side up on a polycarbonate membrane of a Transwell 0.4 µm cell culture insert (Corning/Greiner, Amsterdam, the Netherlands), and flattened with a polished Pasteur pipette. Any surplus medium was removed with sterile filter paper. The membranes with the retina were placed in wells containing 2 mL of neurobasal-A medium (NBA+; Invitrogen) with 1% Penicillin-Streptomycin-Glutamine, 2 mM L-glutamine and B27 supplement such that the bottom of the membrane touched the solution. No fluid was present in the compartment containing the retina. They were then incubated at 37 °C and the medium replaced twice daily.

### 4.3. Virus Transduction

Lentivirus containing a construct consisting of the human arrestin promotor (HXARR3), enhanced Halorhodopsin (eNpHR) from Natronomonas and Enhanced Yellow Fluorescent Protein (EYFP), LV-HXARR3-eNpHR-EYFP, was injected (2.5 µL; titer: 5730 ng/µL; p24 Elisa kit, ZeptoMetrix Corp., New York, NY, USA) between the two plexiform layers of cultured human retinal explants at four different locations, then the retinas were cultured for at least an additional 48 h before experimentation. 

### 4.4. Histology

Pieces of retina were fixated for 20 min at room temperature in 0.1 M phosphate-buffer (pH 7.4) 4% paraformaldehyde, directly after dissection (T0), and after 4 (T4), 7 (T7) and 10 days (T10) of culturing. After fixation, the retinas were rinsed with 0.1 M phosphate buffer (PB) pH 7.4, cryoprotected at room temperature in phosphate buffered saline (PBS) containing 12.5% sucrose for 10 min and then 25% sucrose for 60 min, embedded in Tissue-Tek^®^ O.C.T.™ compound on a slide and frozen in liquid nitrogen. The 10 µm thick sections were mounted on Vectabound-coated slides, dried and stored in a no-frost freezer at −20 °C. The sections were pre-incubated in 2% normal goat serum (NGS) in PBS for 20 min, then incubated with primary antibody overnight at room temperature in PBS containing 5% NGS (Jackson ImmunoResearch, Cambridge, UK, USA). Primary antibody concentrations are given in Table 2.

After washing 3 × 5 min in PBS, the sections were incubated at 37 °C for 20 min in goat anti-mouse IgG 488 or goat anti-rabbit IgG 488 (Jackson ImmunoResearch) and cover slipped with Vectashield containing Propidium Iodide (Vector, Burlinggame, CA, USA). The sections were observed on a Zeiss LSM 510 Meta (Zeiss, Oberkochen, Germany) and a Leica TCS SP8) (Leica, Wetzlar, Gemany).

### 4.5. Single Cell Electrophysiology

A piece of retina was placed photoreceptor side up in a recording chamber (RC27L, Warner Instruments, Hamden, CT, USA) and superfused with Ames’ medium (Sigma, sSt Louis, MI, USA) at 37 °C. The recording chamber was mounted on a Nikon FN600 (Tokyo, Japan) equipped with IR illumination and a CCD camera (Philips, The Netherlands). Electrodes were made from borosilicate glass (GC150TF-10, Harvard Apparatus Ltd., Holliston, MA, USA), pulled to 3–6 MΩ using a Brown Flaming Puller (Model P-87; Sutter Instrument, Novato, CA, USA), and filled with a solution containing 13 mM KCl, 82.4 mM K-Gluconate, 1 mM MgCl_2_, 0.1 mM CaCl_2_, 1 mM EGTA, 10 mM HEPES, 10 mM ATP-K_2_, 1 mM GTP-Na_3_, 20 mM phosphocreatine-Na_2_ and creatine phosphokinase 1.54 mg/100 mL. Electrodes were mounted on a MP-85 Huxley/Wall-type micromanipulator (Sutter Instrument, Novato, CA, USA) and whole cell recordings were made using a DAGAN 3900A integrating patch clamp amplifier (Dagan Corporation, Minneapolis, MN, USA), a 1401 CED AD/DA converter (Cambridge electronics, Cambridge, UK) and a PC running Signal (Cambridge Electronics, Cambridge, UK). Leak currents were estimated by linear extrapolation using the whole cell current occurring immediately after the capacitance transient, but prior to I_h_ activation, for voltage steps from −70 mV to −45 mV (5 mV steps). In each case R^2^ > 0.99.

#### Light Stimuli

The light stimulator consisted of two homemade LED stimulators based on a three-wavelength high-intensity LED (Atlas, Lamina Ceramics Inc., Westhampton, NJ, USA). The peak wavelengths of the LEDs were 624, 525 and 465 nm, respectively, with bandwidths smaller than 25 nm. An optical feedback loop ensured linearity. The output of the LEDs was coupled to the microscope via light guides. White light consisted of equal quantal output of the three LEDs. Two stimuli were used: a spot and a full-field. The spot had a diameter of 20 µm when projected through the 40x water immersion objective (N.A.: 0.55) of the microscope. The full-field stimulus had a diameter of 4500 µm when projected through the microscope condenser (N.A.: 1.25). The three colour channels were calibrated such that they had equal quantal output. The maximal intensity was 3.8 × 10^15^ quanta/s/m^2^. For white light stimulation the halogen lamp of the microscope was used. The maximal intensity of that channel was 850 µW/mm^2^.

### 4.6. Multielectrode Recordings of GCs

After 48 h of culturing, transduced retinal pieces with post-mortem times <20 h were placed photoreceptor side up on a perforated 60 electrode array (60pMEA200/30iR-Ti using a MEA2100 system: Multichannel systems, Reutlingen, Germany) in a recording chamber continuously superfused with oxygenated Ames’ medium (37 °C) mounted on a Nikon Optiphot-2 upright microscope and viewed under IR with an Olympus 2× objective and video camera (Abus TVCC 20530). Extracellular multiunit GC activity was recorded at 25 kHz in MC rack (Multichannel systems, Reutlingen, Germany), zero-phase bandpass filtered (250–6250 Hz) with a fourth-order Butterworth filter in Matlab (MathWorks, Natick, MA, USA), and sorted into single-unit activity with “offline spike sorter” (Plexon, Dallas, TX, USA). Spikes were detected using an amplitude threshold *>* 5 σ_n_ where σ_n_ is an estimation of the background noise

(1) $$\sigma_{n}=median\left\{\frac{\left|x\right|}{0.6745}\right\}$$

with *x* being the bandpass-filtered signal [71]. The detected spikes were manually sorted into single units based on principal components.

#### Light Stimuli

Light responses were assessed by stimulation with a full-field light stimulus presented for 500 ms with a frequency of 0.5 Hz. Light stimuli were generated with Psychophysics Toolbox Version 3 [72] and projected onto the photoreceptors by a DLP projector (Light Crafter 4500, Wintech, Carlsbad, CA, USA) using a custom-built 2× water immersion objective. Only white light stimuli were used with a maximal intensity of 44 μW/mm^2^.

### 4.7. Statistics

The data are presented as mean ± standard error of the mean (SEM). 2-tailed *t*-tests were used to test the significance. Where the mean difference between groups is given, the accompanying *SEM* was determined using the Satterhwaite approximation of *SEM* where *SD_x_* and *n_x_* are the standard deviation and sample size of group *x*.

(2) $$SEM=\sqrt{\frac{SD_{1}^{2}}{n_{1}}+\frac{SD_{2}^{2}}{n_{2}}}$$

## 5. Conclusions

In this paper, we showed that the cultured human retina is a useful model system. However, its scarcity often makes it difficult to obtain a sufficient sample size. Nevertheless, we are convinced that the cultured human retina model is suitable for a variety of research purposes and has the potential to become an essential step in translational research. With continued procedural developments and greater community support regarding tissue availability, it could even help reduce the use of laboratory animals.

## Figures and Tables

**Table 1:** Information regarding the donors whose generosity enabled this study. Details are listed according to the figure associated with the experiments each donor’s tissue was used in and include their age at time of death, the post-mortem time (PTM) before culturing, and additional medical details when they were known. CoD: Cause of Death, CVA: Cerebrovascular accident, NR: Not Reported.

Associated Figure	Age (Years)	PMT (h)	CoD, Agonal State, Medical Conditions/Co-Morbidities (If Known)
Figure 1 and Figure 2	72	17	Mental disorder
	60	29	Pulmonary disease
Figure 3b and Figure 5d,e	78	22	Heart disease
	78	28	CVA, malignancy
	74	25	CVA, malignancy
Figure 3c	59	17	Malignancy
	44	38	Trauma capitis
	59	29	Heart disease
Figure 3d	79	15	Malignancy
	75	6	Malignancy
	41	8	CVA
	41	34	Heart disease
Figure 4a	60	29	Pulmonary disease
	51	28	Pulmonary disease
	69	26	Malignancy
Figure 4b	51	28	Heart disease
	47	31	Malignancy
	67	10	Malignancy
Figure 5b,c	58	19	Mental disorder
	73	20	Mental disorder
Figure 5g	72	18	NR

**Table 2:** Primary antibody concentrations.

Antibody	Dilution	Origin
cleaved caspase-3 rabbit mAb	1:250	Cell Signaling D-175
rabbit anti opsin red/green	1:100	Chemicon AB5405
rabbit anti opsin blue	1:100	Chemicon AB5407
rabbit anti rhodopsin	1:100	Chemicon AB9279
mouse monoclonal anti synaptophysin	1:200	Sigma-Aldrich Clone SVP-38 S5768
mouse anti ribeye	1:500	Transduction laboratories
rabbit anti mGluR6	1:5000	Gift from Dr. Vardi
mouse anti Go-α	1:1000	Chemicon: MAB3073
mouse anti tyrosine hydroxlase	1:200	Chemicon: MAB31

## Abbreviations

BC	bipolar cell
CoD	Cause of death
CVA	Cerebrovascular accident
dLGN	dorsal lateral geniculate nucleus
eNpHR	halorhodopsin
EYFP	enhanced yellow fluorescent protein
GCL	ganglion cell layer
GC	ganglion cell
HC	horizontal cell
HXARR3	human arrestin promotor
INL	inner nuclear layer
IPL	inner plexiform layer
KA	kainic acid
MEA	multielectrode array
mGluR6	metabotropic glutamate receptor
NGS	normal goat serum
NR	Not reported
ONL	outer nuclear layer
OPL	outer plexiform layer
PBS	phosphate buffered saline
PMT	Post-mortem time
RP	retinitis pigmentosa
SEM	standard error in the mean
TH	tyrosine hydroxylases
TTX	tetrodotoxin
